# An Update on Renal Artery Denervation and Its Clinical Impact on Hypertensive Disease

**DOI:** 10.1155/2015/607079

**Published:** 2015-10-01

**Authors:** Aditya Bhat, Ye Min Kuang, Gary C. H. Gan, David Burgess, Alan Robert Denniss

**Affiliations:** Department of Cardiology, Blacktown Mount Druitt Hospital, Blacktown Road, Blacktown, NSW 2148, Australia

## Abstract

Hypertension is a globally prevalent condition, with a heavy clinical and economic burden. It is the predominant risk factor for premature cardiovascular and cerebrovascular disease, and is associated with a variety of clinical disorders including stroke, congestive cardiac failure, ischaemic heart disease, chronic renal failure, and peripheral arterial disease. A significant subset of hypertensive patients have resistant hypertensive disease. In this group of patients, catheter-based renal artery denervation has emerged as a potential therapy, with favourable clinical efficacy and safety in early trials. Additional benefits of this therapy are also being identified and include effects on left ventricular remodeling, cardiac performance, and symptom status in congestive cardiac failure. Utility of renal denervation for the management of resistant hypertension, however, has become controversial since the release of the Symplicity HTN-3 trial, the first large-scale blinded randomised study investigating the efficacy and safety of renal artery denervation. The aim of this paper is to evaluate the history, utility, and clinical efficacy of renal artery denervation technology, including an in-depth appraisal of the current literature and principal trials.

## 1. Introduction

Hypertension is a clinical disorder that is defined as an aberrancy of blood pressure, with systolic blood pressure (SBP) equal to or greater than 140 mmHg and/or diastolic blood pressure (DBP) equal to or greater than 90 mmHg [1]. It is the most common chronic disease in developed societies and is associated with a variety of clinical disorders including stroke, congestive cardiac failure, chronic renal failure, ischaemic heart disease, and peripheral arterial disease [2, 3].

As blood pressure is a continuous variable, hypertension can be categorized into separate classes or stages, with current clinical classification systems encompassing Stages 1 (SBP 140–159 mmHg and/or DBP 90–99 mmHg), 2 (SBP 160–179 mmHg and/or DBP 100–109 mmHg), and 3 (SBP ≥ 180 mmHg and/or DBP ≥ 110 mmHg) [4]. These increments are divided as they provide prognostic value [5].

Poorly controlled hypertension is the predominant risk factor for premature cardiovascular disease, with an estimated contribution of 54% in all cerebrovascular accidents (CVAs) and 47% of all coronary events globally [6]. These risks, primarily attributable to accelerated atherogenesis and increased arterial impedance, have been found with elevations in SBP or pulse pressure in persons over the age of 60, and elevations in DBP in younger individuals [7].

Current therapeutic strategies in the management of hypertension are based on lifestyle interventions and pharmacological agents, with studies showing significant reductions in blood pressure (10–12 mmHg SBP, 5-6 mmHg DBP) to be associated with an overall decreased morbidity and mortality, including an estimated 38% risk reduction of CVA and 16% risk reduction of coronary artery disease [8]. In line with studies showing diminishing returns associated with aggressive blood pressure reduction below SBP of 140 mmHg, current treatment guidelines advocate for a uniform approach with suggested targeting of SBP to less than 140 mmHg for all hypertensive groups, regardless of cardiovascular risk profiles [9, 10].

Despite the obvious benefits as well as the wide range of available pharmaceutics today, the management of hypertension remains unsatisfactory even in industrialised nations. While this is primarily attributable to therapeutic inertia and poor adherence, an increasing proportion of patients are now recognized to have refractory or resistant hypertension, with an almost threefold increase in cardiovascular risk in this group [11].

## 2. Aetiology, Classification, and Pathophysiology

Hypertension can be classified according to aetiology, with main subdivisions delineating primary and secondary forms. Primary hypertension, commonly referred to as essential hypertension, accounts for the majority of cases of hypertensive disease (>95%) [12]. It is a multifactorial disorder that has a strong heritable component with complex genetic and environmental interactions in which sympathetic overactivity and renal pathology form a large constituent of the pathogenesis [12–15]. Secondary hypertension, in contrast, embodies a small proportion of patients with diagnosed hypertension. It is caused by a variety of medical and medication-related conditions and disease states [12].

The pathophysiology of hypertension is complex. Under normal physiological processes, the renal system provides signals to the central nervous system to regulate whole body vascular resistance. These afferent and efferent pathways regulate blood pressure at multiple levels including the renin-angiotensin-aldosterone system, local nephron blood flow, and transporter regulation of sodium and water excretion [16]. A synergistic effect is further produced by the autonomic nervous system. Via actions on the heart, blood vasculature, and kidney, the sympathetic nervous system causes increases in cardiac output, vascular resistance, and sodium and fluid retention [13, 14].

In hypertensive individuals, high sympathetic drive accompanied by suppressed parasympathetic action contributes to hypertensive disease [17]. In addition, peripheral and central arterial baroreceptors are reset at higher thresholds in this group [18], leading to maintenance of higher mean arterial pressures, irrespective of intravascular volume. Compared to normotensive individuals, patients with hypertension also have an enhanced chemoreflex system, with heightened renal sympathetic stimulation and greater vasoconstrictor responses to noradrenaline [19]. With the increased activation of renal sympathetic function, increased spillover of noradrenaline ensues, resulting in augmentation of renin secretion from the kidney. The consequent increase in sodium and fluid retention, renal artery vasoconstriction, and decreased renal perfusion further perpetuates the hypertensive state, worsening renal perfusion and thus leading to a vicious cycle [20–22].

## 3. Resistant Hypertension

A subset of individuals with hypertensive disease are characterized as having resistant hypertension. As per The Joint National Committee 7 consensus, resistant hypertension is defined as a SBP at or exceeding 140 mmHg and/or a DBP at or exceeding 90 mmHg despite full compliance to the maximum tolerated dose of 3 or more antihypertensive medications, including a diuretic [1]. Resistant hypertension is not to be confused with poorly controlled hypertension or “pseudo-hypertension,” a condition that is attributed to poor adherence, suboptimal medication regime, or secondary hypertension and which does not represent true treatment resistance [23].

To date, no large prospective studies are available to substantiate the prevalence of true resistant hypertension. Data extrapolated from small studies have established the prevalence ranging from 5 to 20% of cases [24]. It is acknowledged however that, for those diagnosed with resistant hypertension, therapeutic options are limited and patients are subject to an almost threefold increase in cardiovascular risk compared to those with controlled hypertension, conveying the need for alternative therapeutic options in blood pressure control beyond pharmacological strategies [25].

## 4. Renal Artery Denervation: Concept

Renal sympathetic nerves, afferent and efferent, are embodied within the wall of the renal artery and are required for maintenance of systemic hypertension [13, 14, 16]. The concept of sympathetic nerve modulation as a management tool for systemic hypertension is not new, with surgical intervention being used prior to the advent of pharmacotherapeutics.

Surgical resection of thoracic, abdominal, and pelvic sympathetic nerves has been utilised in the past for the management of Stage 3 hypertension. Despite sustained blood pressure control, these methods were associated with high perioperative mortality, and long-term deleterious effects including significant dysfunction of organs (bladder, bowel, and genitals) supplied by these nerves [26].

Given the efficacy of sympathetic modulation, the concept of selective disruption of sympathetic nerve supply was hypothesized. The idea was that, via percutaneous approach, a catheter can be inserted via the femoral artery and placed into the major renal artery on each side to deliver radio-frequency (RF) energy to the adventitia of the vessel, one of the layers of the arterial wall which houses the renal sympathetic and afferent nerves. The primary theoretical benefit would be sustained reduction of blood pressure, brought about by disruption of primary sympathetic output, without leading to the generalized adverse effects of broader sympathetic disruption.

## 5. Renal Artery Denervation: Clinical Trials

Percutaneous transcatheter renal artery denervation was first explored in the early 2000s. Regarded as a radical approach to hypertension, the idea was initially met with skepticism by most when first introduced. This perception persisted despite animal trials showing a significant reduction in blood pressure and renal noradrenaline content comparable to direct surgical renal denervation with minor procedural related complications [27–29].

### 5.1. Symplicity HTN-1 and HTN-2

The first human trial investigating the efficacy of catheter-based renal artery denervation was the Catheter-Based Renal Sympathetic Denervation for Resistant Hypertension (Symplicity HTN-1) Trial (2011). In this multicentre, nonrandomised study, the investigators looked at primary endpoints of safety and sustained blood pressure reduction from the procedure, with secondary endpoints of procedural effect on renal noradrenaline spillover and effect on renal function [30]. Inclusionary criteria were that of resistant hypertension (SBP ≥ 160 mmHg) despite three antihypertensive medications (including a diuretic) in the absence of haemodynamically significant valvular disease or renovascular abnormalities determined via angiographic assessment.

**Figure 1:**
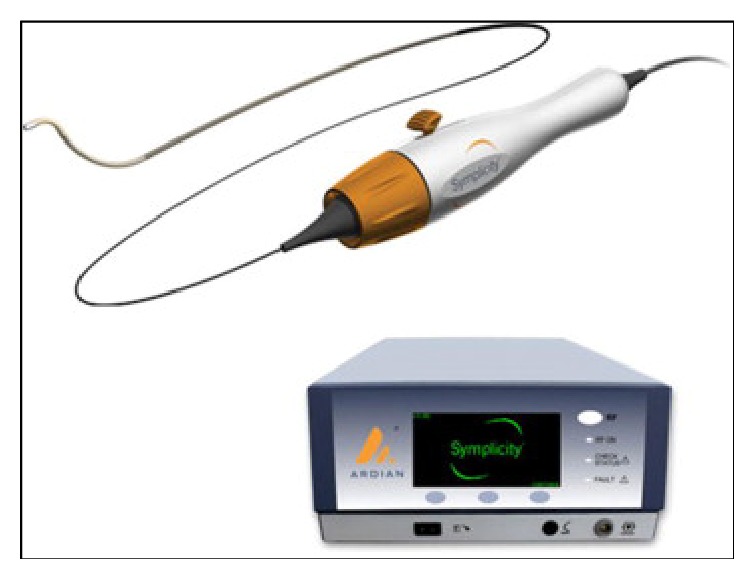
Symplicity catheter system.

**Figure 2:**
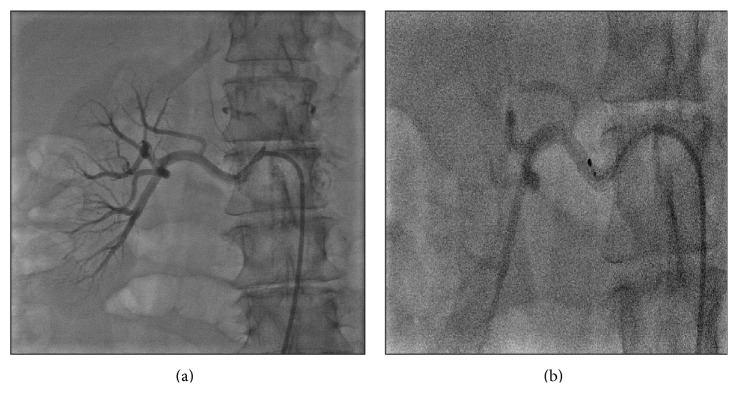
Catheter-based renal artery denervation procedure.

Patients undergoing the procedure were commenced on low dose Aspirin (100 mg daily) a week prior to the procedure with intravenous heparin cover during the procedure itself. RF ablation was performed with the Medtronic Simplicity Catheter (Figure 1) via a 6Fr or 8Fr guide. Four-to-eight ablations were delivered within each renal artery, each lasting approximately 2 minutes, and were separated both longitudinally and rotationally within the length of the artery (Figure 2). Median time of procedure from first to last RF ablation delivery was 38 minutes. After procedure, renal angiography was performed to identify any irregularities or stenosis. Patients were subsequently followed up at 1, 3, 6, 9, and 12 months with average office-based blood pressure measurements [30].

A total of 153 patients (60 females, 93 males), who were on average taking 5.1 antihypertensive medications, with a mean SBP and DBP of 177 mmHg and 98 mmHg (±17/14 mmHg), respectively, were included into this pilot study. Renal artery denervation was performed in 149 (97%) patients without complications. In the remaining 4 patients, renal artery dissection occurred in one patient prior to delivery of RF ablation that was treated with a renal artery stent without any further sequelae, whilst the other 3 patients developed a pseudo-aneurysm of the femoral access site that was managed conservatively with monitoring and analgesia [30]. Intraprocedural diffuse abdominal pains were reported in all cases that were managed with intravenous narcotics and anxiolytics. In terms of renovascular safety, repeat renal imaging that was performed in 81 patients at 6 months did not show any new abnormalities or stenosis in the treated arteries. One patient was noted to have progression of preexisting renal artery stenosis in the proximal portion of the renal artery, distant to the sites of RF ablation. This was treated successfully with a renal artery stent. There was no deterioration in renal function in the entire cohort [30].

Overall, the investigators found transcatheter renal artery denervation to be effective in the treatment of resistant hypertension. 92% of patients had significant (defined as a reduction in SBP of ≥ 10 mmHg) office-based blood pressure reductions at 1 month following procedure that was sustained to the 24-month follow-up period (average SBP reduction of 32 mmHg, average DBP reduction of 14 mmHg at 12 months following procedure) in the treated cohort. SBP and DBP were noted to be significantly lower than baseline readings at all time-points after procedure with the exception of DBP readings at 12 months [30]. Improvements in blood pressure dipping patterns were also observed with the procedure. Prior to treatment, 67% of the treated cohorts were either nondippers or reverse-dippers. This was noted to reduce to 33% after procedure. Importantly, there was no significant deterioration in renal function and a reduction in renal noradrenaline spillover was noted concurrently with the achieved blood pressure response [30].

A 3-year follow-up in a cohort of 88 (of the original 153 patients) showed that the reductions in blood pressure were noted to persist throughout 36 months, with an average blood pressure reduction of −32/−14 mmHg (*p* < 0.01). Of this group, approximately 50% achieved the goal of a SBP <140 mmHg. A drop in SBP of ≥10 mmHg was seen in 85% of the cohort at 12 months and 93% at 36 months. Furthermore, the proportion of patients with a SBP of 180 mmHg or higher had notably decreased from 30% at baseline to 5% at 36 months. One new renal artery stenosis was reported at 24 months, which was managed successfully with renal arterial stenting, and three deaths unrelated to the procedure were noted during this follow-up period [31]. The results were encouraging, highlighting the efficacy and safety of the procedure and alluding to the lack of functional reinnervation of the kidney over a longer time-frame [31].

Following the success of the Symplicity HTN-1 trial, a second trial was undertaken to evaluate the efficacy of transcatheter renal artery denervation within a randomised cohort. Titled the Renal Sympathetic Denervation in Patients with Treatment Resistant Hypertension or Symplicity HTN-2, the trial essentially mirrored its brother trial in terms of methodology but with the addition of a randomised (but nonblinded) control arm. The primary endpoint of the trial was the between-group change in average office-based measurements of SBP from baseline to 6 months following randomization. Secondary endpoints were of procedural safety, composite of cardiovascular endpoints, and additional measurements of blood pressure reduction at 6 months after randomization [27].

After anatomical screening of the renal artery to confirm eligibility, patients were randomly assigned to the interventional group to undergo catheter-based renal denervation or to a control group, which were isolated to medical therapy only. All patients assigned to the interventional cohort were administered heparin cover intraprocedurally and RF ablation was performed with the Medtronic Symplicity Catheter with the same technique. For both interventional and control groups, changes to baseline doses of antihypertensive therapy were advised against unless deemed medically necessary. All patients were followed up at 1, 3, and 6 months following procedure for office-based blood pressure assessment [32].

A total of 106 patients (45 females, 61 males) were included into the study, 52 of whom were allocated to the denervation group and 54 to the control group. Baseline characteristics of patients in both groups did not differ significantly in terms of age, sex, race, baseline blood pressure, and length of antihypertensive therapy [32]. With regard to the primary endpoint, catheter-based renal artery denervation was associated with a significant reduction in blood pressure compared to the control group. At 6 months after randomisation, office-based measurements of blood pressure in the renal denervation group were significantly reduced compared to baseline, a benefit that was reproducible by concordant measurements of home blood pressure and 24-hour ambulatory blood pressure monitoring. Of the treated cohort, 84% had >10 mmHg reduction in SBP, 80% had SBP values below 160 mmHg, and 40% had SBP values below 140 mmHg. Only 10% of the patients had no reduction in SBP. In line with the reduction of blood pressure, a greater reduction in urine albumin-to-creatinine ratio was also observed with the treated cohort [32].

No serious procedural complications were reported after procedure. Seven of 52 patients developed transient intraprocedural bradycardia that resolved with atropine. One femoral artery pseudo-aneurysm and one postprocedural hypotension were reported which was managed with manual compression and reduction in antihypertensive therapy, respectively. No postprocedural renal artery stenosis or aneurysms were noted in the 43 patients (37 renal duplex imaging procedures, 5 MRI procedures, and 5 CT angiographies) receiving renal imaging at 6 months. No deterioration in renal function was noted from baseline in both groups at 6 months [32].

A retrospective cost-benefit analysis of the Symplicity HTN-2 trial was performed by Geisler et al. (2012), which concluded that, given the robust sustained reductions in blood pressure, extrapolation to lifetime clinical probabilities (including CVA, myocardial infarction, all coronary artery diseases, heart failure, and end-stage renal failure) revealed an improvement in median survival for patients undergoing renal artery denervation compared to standard therapy (18.4 years versus 17.1 years). This benefit extrapolated to a cost-saving of 31,460 USD per quality-adjusted life-year, alluding to long-term savings in the face of high short-term costs [33].

All in all, the results of both Symplicity trials highlighted the clinical efficacy and safety of catheter-based renal artery denervation in resistant hypertension. Despite the promising results however, both trials were not without limitations that exceeded just the relatively small employed sample sizes.

Of note was the potential bias in trial design. The Symplicity HTN-1 trial was a “proof-of-principle” study that was unblinded allowing possible selection bias in recruitment of patients and observer bias in the measurements of office-based blood pressure. Though randomised, investigators in the Symplicity HTN-2 Trial were not blinded to the treatment modality and no sham procedures were performed in the control group, similarly raising the possibility of observer bias with the measurement of blood pressure. In both trials, patients with moderate-to-severe renal impairment were excluded, discounting any definitive statements regarding its efficacy in this population. Moreover, only patients with favourable renal artery anatomy to catheter-based renal artery denervation were included into both trials, emphasizing the prospect of dissimilar efficacy and procedural safety in patients with less favourable anatomy undergoing the procedure.

Though office blood pressure measurements were substantiated with ambulatory blood pressure measurements in both trials, they were limited to a small subset of participants and were used primarily to confirm the blood pressure lowering effect of the procedure, casting doubts as to the exact blood pressure response after procedure. Furthermore, the causes of response variability as seen in the 10% nonresponders in the Symplicity HTN-2 trial have not yet been well elucidated, with queries of incomplete denervation or sympathetic reinnervation foremost in mind. From an operator perspective, the absence of an objective measure of procedural success was also seen as problematic.

### 5.2. The EnligHTN I Trial

**Figure 3:**
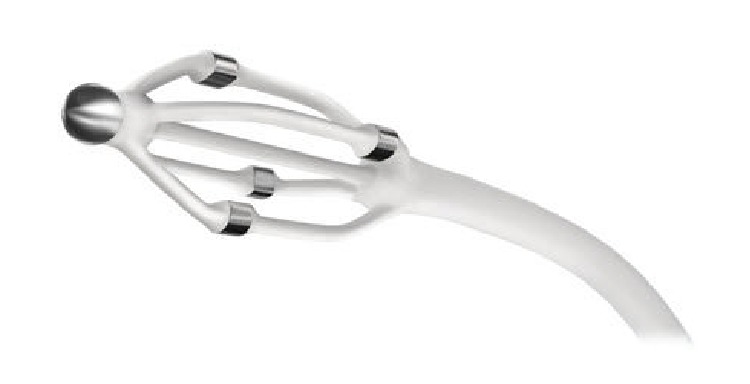
EnligHTN catheter system.

The EnligHTN I trial was a prospective multicentre, nonrandomised, “first-in-human” trial of the multielectrode EnligHTN catheter. The primary efficacy endpoint of the trial looked at the reduction of office BP measurements following denervation procedure at 6 months compared with baseline. Primary safety endpoints were all adverse events that occurred during the study period. Unlike the Symplicity catheter, the EnligHTN catheter (St. Jude Medical) (Figure 3) consisted of an expandable electrode basket housing four Platinum-Iridium-based electrodes. Each electrode was capable of delivering low-level RF ablations to the renal arterial wall and the expandable feature of the basket allowed for establishment of better apposition of electrodes in reference to potential ablation sites on the vessel wall.

Recruitment in this study included patients in varying age brackets (18–80 years of age) who were selected by referral from primary healthcare providers or specialists across four participating centres. Enrolled subjects were required to have a clinic-based SBP measurement of ≥160 mmHg (≥150 mmHg for diabetics) despite prolonged use of at least three antihypertensives (including a diuretic) and suitable renal artery anatomy assessed via renal artery angiography. Participants with small (≤4 mm diameter, ≤20 mm length) [*n* = 1], multiple [*n* = 2], or highly tortuous renal arteries [*n* = 0] were excluded from the procedure as were those with significant (>30%) renal artery stenosis [*n* = 4] [34]. All eligible participants had 24-hour ambulatory blood pressure monitoring with strict compliance to regular antihypertensive regime for at least two weeks prior to enrolment. After this period, participants then had a complete baseline assessment which included collection of basic biochemistry (full blood count, serum creatinine, estimated glomerular filtration rate (eGFR), cystatin C) and urine analysis (urine albumin-to-creatinine ratio) [34].

Renal artery denervation procedure was performed under conscious sedation and local anaesthesia [34]. A minimum of four and maximum of eight ablation sites were performed in each main renal artery in a circumferential pattern, with each ablation lasting 90 seconds. Patients were monitored postprocedurally, with scheduled follow-up visits at 1, 3, and 6 months, and continuing to 24 months. Renal arterial imaging via computed tomography and duplex ultrasonography was repeated at 6 months [34].

A total of 46 (15 females, 31 males) patients were included into the study and underwent the renal artery denervation procedure. With regard to the primary efficacy outcome, investigators of the trial found significant blood pressure reductions with the renal artery denervation procedure performed with the EnligHTN catheters compared to baseline. At 1, 3, and 6 months, average blood pressure reductions of −28/10 mmHg, −27/10 mmHg, and −26/10 mmHg (*p* < 0.0001) were observed, respectively. The blood pressure reductions were sustained to the 18-month period, whereby an average SBP reduction of 24 mmHg was noted, with the majority (77%) of studied participants having a clinically significant response to therapy. In terms of safety, no serious adverse events were noted in the cohort. A nonclinically significant reduction in eGFR was reported at 6 months (baseline 87 ± 19 mL/min/1.73 m^2^; at 6 months 82 ± 20 mL/min/1.73 m^2^). At 18 months however, no clinically significant changes to renal function were noted [34, 35].

Like the Symplicity HTN-1 study, the EnligHTN trial was a “proof-of-principle” study that was nonrandomised and unblinded, allowing for possible selection bias in the recruitment of patients and observer bias in the measurements of office-based blood pressure. Despite its drawbacks, the results of the trial were promising, essentially mirroring the findings of both Symplicity HTN trials and further supporting the efficacy and safety of renal artery denervation as a highly effective therapeutic option in resistant hypertension. With the success of these early trials, demand for percutaneous devices rose, driving the development of a wide variety of alternative catheter-based systems for renal artery denervation (see Table 1).

## 6. Renal Artery Denervation: Potential Extended Efficacy

The OLOMOUC I Study (The Effect of Renal Denervation in Patients with Advanced Heart Failure) is an unpublished pilot study by Taborsky et al. investigating the efficacy of renal artery denervation in advanced cardiac failure. In this study, 51 patients with advanced cardiac failure (New York Heart Association (NYHA) Functional Class (FC) III/IV) were randomised to either catheter-based renal denervation plus standard medical therapy or to solitary standard therapy with follow-up over a 12-month period. Primary endpoints of the study looked at left ventricular systolic function calculated by 2D echocardiography and safety profile of the denervation procedure. Secondary endpoints were that of resting heart rate, renal function, NT-proBNP levels, and status of NYHA FC [36]. Inclusionary criteria comprised patients with NYHA FC III and/or IV heart failure who were stable on optimal medical therapy over a 6-month period prior to the intervention, suitable renal artery anatomy, resting heart rate > 70 beats per minute, and a eGFR > 50 mL/min/1.73 m^2^ [36].

Overall, the intervention group saw a modest improvement in left ventricular ejection fraction (LVEF) [mean LVEF 25% at baseline to 31% at twelve months (*p* < 0.01)] relative to the control group [mean LVEF 26% at baseline to 28% at twelve months (*p* = 0.36)]. Other markers of left ventricular impairment (LVESVI, LVEDVI, and NT-proBNP) were similarly improved. In addition to the improvements in left ventricular function, there was a trend towards lower rehospitalisations for heart failure (8 versus 18) in the intervention arm (*p* < 0.001). Two complications, however, were registered in the intervention arm; one patient developed a femoral fistula formation and the second had formation of postoperative thrombus [36].

The findings of this yet-to-be-published trial indicate an additional benefit of denervation therapy, with proposed reduction in neurohormonal substrates for maintenance and progression of cardiac failure. These changes in sympathetic activity, with downstream changes in hormones related to left ventricular remodeling, may represent an additional tool in the management of advanced heart failure with reduced ejection fraction, independent of its effects on afterload reduction.

Another study, published in early 2012, investigated the effect of renal artery denervation therapy on left ventricular parameters, including echocardiographic indices of systolic and diastolic function. This study employed 64 participants, which were either placed into the treatment arm (*n* = 46) or control arm (*n* = 18). Patients over the age of 18 with a clinic-recorded SBP ≥160 mmHg despite management with three antihypertensives (including a diuretic) were included into the study. These subjects were followed over a 6-month period, with transthoracic echocardiography performed at baseline, 1 and 6 months [37].

Echocardiographic endpoints included LVEF, left ventricular mass index (a marker of left ventricular hypertrophy), mean interventricular septal thickness, and mitral inflow parameters (lateral E/E′, isovolumic relaxation time) measured via Doppler echocardiography. Besides a sustained reduction in office-based blood pressure (SBP/DBP −27.8/−8.8 mmHg at 6 months, *p* < 0.001), a significant reduction in markers of diastolic impairment (interventricular septal thickness, left ventricular mass index, mitral valve lateral E/E′, isovolumic relaxation time) was appreciated in the treatment group. Additionally, a statistically significant improvement in LVEF was noted (baseline LVEF: 63.1 ± 8.1% versus 70.1 ± 11.5% at 6 months, *p* < 0.001) [37].

Interestingly, although regression of parameters of left ventricular diastolic dysfunction was incremental with SBP reduction, “nonresponders” (patients which demonstrated <10 mmHg SBP reduction at 6 months following renal artery denervation) still demonstrated a marked reduction in these indices [37]. This finding would support the hypothesis that renal artery denervation causes regression of left ventricular remodeling independent of its effects on blood pressure.

This improvement in cardiac function has been shown to translate to a symptomatic benefit in a recent trial by Davies et al. (2013), which assessed seven patients with chronic heart failure with reduced LVEF receiving renal artery denervation therapy over a six-month period. Of interest, the mean blood pressure on referral was 112/65 mmHg (normotensive range), significantly lower than that employed in the majority of trials [38].

Following denervation, a nonsignificant trend towards blood pressure reduction was found (SBP −7.1 ± 6.9 mmHg, *p* = 0.35; DBP −0.6 ± 4.0 mmHg, *p* = 0.88) at 6 months, with no hypotensive events noted. Renal function was unaffected. All seven patients reported a significant symptomatic improvement, with significant quantitative improvement in six-minute walk distance at 6 months (Δ = 27.1 ± 9.7 m, *p* = 0.03). No procedural or postprocedural complications were noted [38]. The novel finding of this study, in addition to improvement of symptom status, was the lack of haemodynamic instability achieved after renal artery denervation in normotensive patients, which may indicate a secondary compensatory mechanism to maintain SBP within acceptable limits for organ homeostasis despite sympathetic disruption or may indicate nonefficacy of the procedure in blood pressure control.

In addition to the previously mentioned effects on left ventricular remodeling, cardiac performance, and symptom status in congestive cardiac failure, studies have also demonstrated the efficacy of renal denervation in patients with impairment of the baroreflex sensitivity. In a study by Zuern et al. (2013), 50 patients with resistant hypertension and a mean ambulatory SBP of 157 ± 22 mmHg were enrolled in the prospective cohort study and underwent renal denervation therapy. At six-month follow-up subsequent to procedure, 26 patients (52%) achieved a drop in mean ambulatory SBP of ≥10 mmHg. Upon review, impaired baroreflex sensitivity was strongly associated with response to renal denervation (*p* < 0.001) [39].

## 7. The Symplicity HTN-3 Study

The Symplicity HTN-3 study was a multicentre, prospective, double-blinded, randomised study investigating the efficacy and safety of renal arterial denervation using the Symplicity catheter system in patients with medically refractory hypertension. Like the previous Symplicity HTN trials, the primary efficacy endpoint of the study looked at the change in office SBP measurements at 6 months. Secondary efficacy endpoints of the trial differed, looking at the change in mean 24-hour ambulatory SBP. The primary safety endpoint was a composite of major adverse events (defined as death from any cause, end-stage renal disease, an embolic event resulting in end-organ damage, renal artery or other vascular complications, or hypertensive crisis within 30 days or new renal artery stenosis of more than 70% within 6 months) [40].

Inclusionary criteria were that of resistant hypertension (SBP ≥ 160 mmHg) despite three or more maximally tolerated antihypertensive medications (including a diuretic) in the absence of haemodynamically significant valvular disease or renovascular abnormalities determined via angiographic assessment. All recruited patients underwent a confirmatory screening visit beforehand to confirm SBP of >160 mmHg and adherence to medications. Once included, patients were then randomised in 2 : 1 fashion to treatment arm and control arm, respectively. Those in the control group underwent renal angiography only (sham control). Patients in the interventional arm underwent the renal denervation procedure which was performed with the Medtronic Symplicity Catheter [40].

A total of 535 patients (325 males, 210 females) were included into the study, 364 of whom were allocated to the interventional cohort and 171 to the control cohort. Procedural technique and periprocedural pharmacotherapy of the interventional arm were unchanged from the previous Symplicity trials. Regardless of group, all blood pressure assessors were unaware of study group assignments and a blinding index was utilised to verify the effectiveness of blinding at hospital discharge and at 6 months. Patients were subsequently followed up at 6-month intervals after randomisation (with an aim follow-up up to 5 years). Changes to baseline doses of antihypertensive therapy were not encouraged unless deemed medically necessary [40].

In terms of safety, no significant difference was noted in terms of overall composite adverse events between denervation and control groups. The rate of major adverse events in the denervation group was 1.4% compared to 0.6% in the control group (*p* = 0.67). No significant changes in renal function were observed between both groups [40].

With regard to efficacy endpoints however, investigators surprisingly found no clinically significant changes in baseline SBP between both groups. At 6 months, an average office blood pressure reduction of −14.3 ± 23.93 mmHg was noted in the intervention arm compared to −11.74 ± 25.94 mmHg in the control group with a between-group difference of −2.39 mmHg (95% confidence interval [CI], −6.89 to 2.12; *p* = 0.26 with a superiority margin of 5 mmHg) [40]. Ambulatory blood pressure reductions were similarly nonsignificant, with an average reduction of −6.75 ± 15.11 mmHg in the denervation group and −4.79 ± 17.25 mmHg in the control group at 6 months, for a between-group difference of −1.96 mmHg (95% CI, −4.97 to 1.06; *p* = 0.98 with a superiority margin of 2 mmHg). There was also no significant between-group difference in terms of change in heart rate from baseline to 6 months (−3.8 ± 11.2 beats per minute in the denervation group and −2.7 ± 10.9 beats per minute in the sham-procedure group; *p* = 0.30) [40].

Though the trial essentially confirmed the safety of the procedure, the negative findings in terms of efficacy were sobering and essentially contradicted the findings of the other Symplicity HTN and denervation trials.

Investigators and stakeholders raise several possibilities to explain the discrepancy of the findings. Of note was the difference in population studied. Whilst there were no significant differences in terms of baseline characteristics between the two arms in the Symplicity HTN-3 trial, the trial included a significant number of African Americans (90 patients in the intervention arm, 50 patients in the control group), a demographic that was not present in the previous Symplicity trials. Subgroup analysis of this demographic showed a paradoxical effect of denervation therapy, with preferential (albeit not statistically significant) blood pressure lowering effects in the control arm as opposed to the Caucasian cohort [41, 42].

Patient characteristics and medication profiles between the Symplicity HTN-2 and Symplicity HTN-3 trials likewise differed, with a higher proportion of obese patients, patients with increased cardiovascular risk factors, and patients with greater use of diuretics and aldosterone antagonists as part of their antihypertensive regimen included into the Symplicity HTN-3 trial. In addition, participants included in the trial only received maximal antihypertensive therapy for two weeks prior to evaluation of efficacy, whilst a recommendation for at least two months is endorsed by current guidelines on hypertension, raising the possibility that patients with an incorrect diagnosis of resistant hypertension were included into the trial [43].

From a procedural and technical perspective, a large proportion of operators in the Symplicity HTN-3 trial had no previous experience with the procedure and as such may have been less experienced compared to the site-specific trained Symplicity HTN-1 and -2 operators. Moreover, whilst indirect electrical impedence was utilised to discern contact with the arterial wall and thus guide the positioning of the catheters, there were unfortunately no objective measures of procedural success. As such, inadequate thermoablation was possible, regardless of the experience of the operator [41, 44].

Finally, in the Symplicity HTN-3 trial, there were tighter entry control criteria with regard to ambulatory blood pressure. As part of the inclusionary criteria, ambulatory blood pressure monitoring was performed with the aim of excluding patients with pseudo- or white-coat hypertension. The same did not hold true for the Symplicity HTN-2 trial. This may have led to overestimation of initial blood pressure measurements in the earlier Symplicity trials and thus led to lower follow-up blood pressure results.

## 8. Conclusion

Given the heavy clinical and economic burdens of hypertensive disease, new treatment methodologies are currently being explored. Catheter-based renal artery denervation technology is one of the novel treatments in the management of resistant hypertension. Based on the concept of sympathetic nerve modulation, many were initially in favor of the technology given the promising results of the early trials. In view of the disheartening results of the Symplicity HTN-3 trial however, widespread utilisation of the procedure has now become controversial.

Skeptics allude to the lack of compelling evidence regarding efficacy in the trials and the significant profit driven goals with the technology. Advocates on the other hand argue that further vigorous randomised trials are required before complete disbanding of the technology and state that while the use of renal artery denervation in blood pressure reduction may be contentious, the efficacy of the technology in other areas is yet unproven and potentially beneficial.

As smaller studies have shown benefit in left ventricular remodeling, cardiac performance, and symptom status in patients with cardiac failure when utilized as an accessory tool to medical therapy, larger trials are recommended to assess the effect of this modality in this group. Additional double-blinded trials would also be recommended in patients with impaired baroreceptor sensitivity, as this represents a potentially vulnerable group of sympathetic hypertensive diseases which may be amenable to percutaneous catheter renal denervation therapy.

## Figures and Tables

**Table 1:** Renal denervation catheter systems.

Catheter type	BSC Vessix	MDT Symplicity	MDT Spyral	STJ EnligHTN	COV OneShot	ReCor Gen-2 Paradise	JNJ Thermo-Cool
Picture	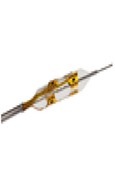	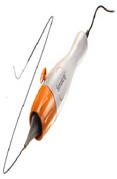	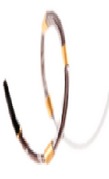	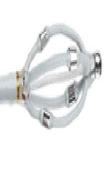	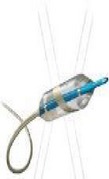	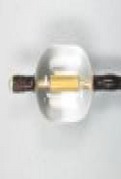	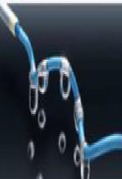

Catheter design	Balloon catheter 4–8 electrodes	Catheter with single electrode	Pigtail catheter 4 electrodes	Basket with four electrodes	Balloon catheter helical electrode and cooling	Balloon catheter, internal cooling	Pigtail catheter with 5 electrodes and cooling

Energy	Bipolar RF	Monopolar RF	Monopolar RF	Monopolar RF	Monopolar RF	Ultrasound	Monopolar RF

Power	~1 W	8 W	8 W	6 W	25 W	~12 W	15 W
